# Supplementary material for: Strong Sex-Dependent Effects of Malnutrition on Life- and Healthspan in Drosophila melanogaster
Source: Insects. 2023 Dec 26;15(1):9. doi: 10.3390/insects15010009 (PMC10816799; doi:10.3390/insects15010009)
Supplement: Supplementary file 1 [file insects-15-00009-s001.zip › Supplementary Material 1.pdf]

SUPPLEMENTARY MATERIAL FOR

Strong Sex-Dependent Effects of Malnutrition on Life- and Healthspan in *Drosophila melanogaster*

Nikolaj Klausholt Bak [1], Palle Duun Rohde [2] and Torsten Nygaard Kristensen [1,*]

[1] Department of Chemistry and Bioscience, Aalborg University, Frederik Bajers Vej 7H, DK 9220 Aalborg, Denmark; ; ndb@bio.aau.dk

[2] Department of Health Science and Technology, Aalborg University, Selma Lagerløfs Vej 249, DK 9260 Gistrup, Denmark

[*] Correspondence: tnk@bio.aau.dk; Tel.: +45 6146 3375

This supplementary file contains:

Supplementary Figures

Figure S1	Treatment of male flies before dry weight measurement
Figure S2	Forrest plot of Tukey HSD on female dry weight
Figure S3	Forrest plot of Tukey HSD on male dry weight
Figure S4	Forrest plot of Tukey HSD on female lipid content
Figure S5	Forrest plot of Tukey HSD on male lipid content
Figure S6	Forrest plot of Tukey HSD on female locomotor activity
Figure S7	Forrest plot of Tukey HSD on male locomotor activity
Figure S8	Forrest plot of Tukey HSD on female HKDT
Figure S9	Forrest plot of Tukey HSD on male HKDT
Figure S10	Forrest plot of Tukey HSD on female fitness score
Figure S11	Forrest plot of Tukey HSD on male fitness score

Supplementary Tables

Table S9	Summary of Multivariate Cox regression on lifespan
Table S10	Summary of ANOVA on dry weight
Table S11	Summary of ANOVA on dry weight of female and male flies separately
Table S12	Summary of ANOVA on lipid content
Table S13	Summary of ANOVA on lipid content of female and male flies separately
Table S14	Summary of ANOVA on locomotor activity
Table S15	Summary of ANOVA on locomotor activity of female and male flies separately
Table S16	Summary of ANOVA on HKDT
Table S17	Summary of ANOVA on HKDT of female and male flies separately
Table S18	Summary of ANOVA on fitness score
Table S19	Summary of ANOVA on fitness score of female and male flies separately

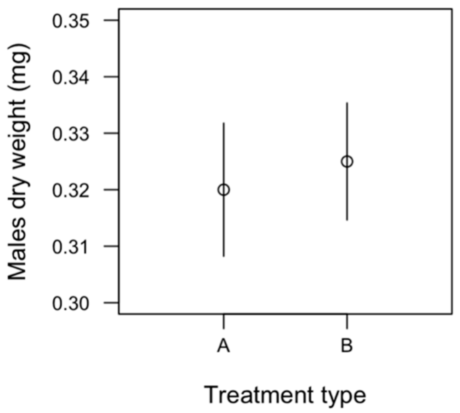

Figure S1. The effects of treatment A and B on dry weight of male flies. Flies on treatment A is the control group. Flies on treatment B were exposed to 2 hours of heat stress assay at 39°C.

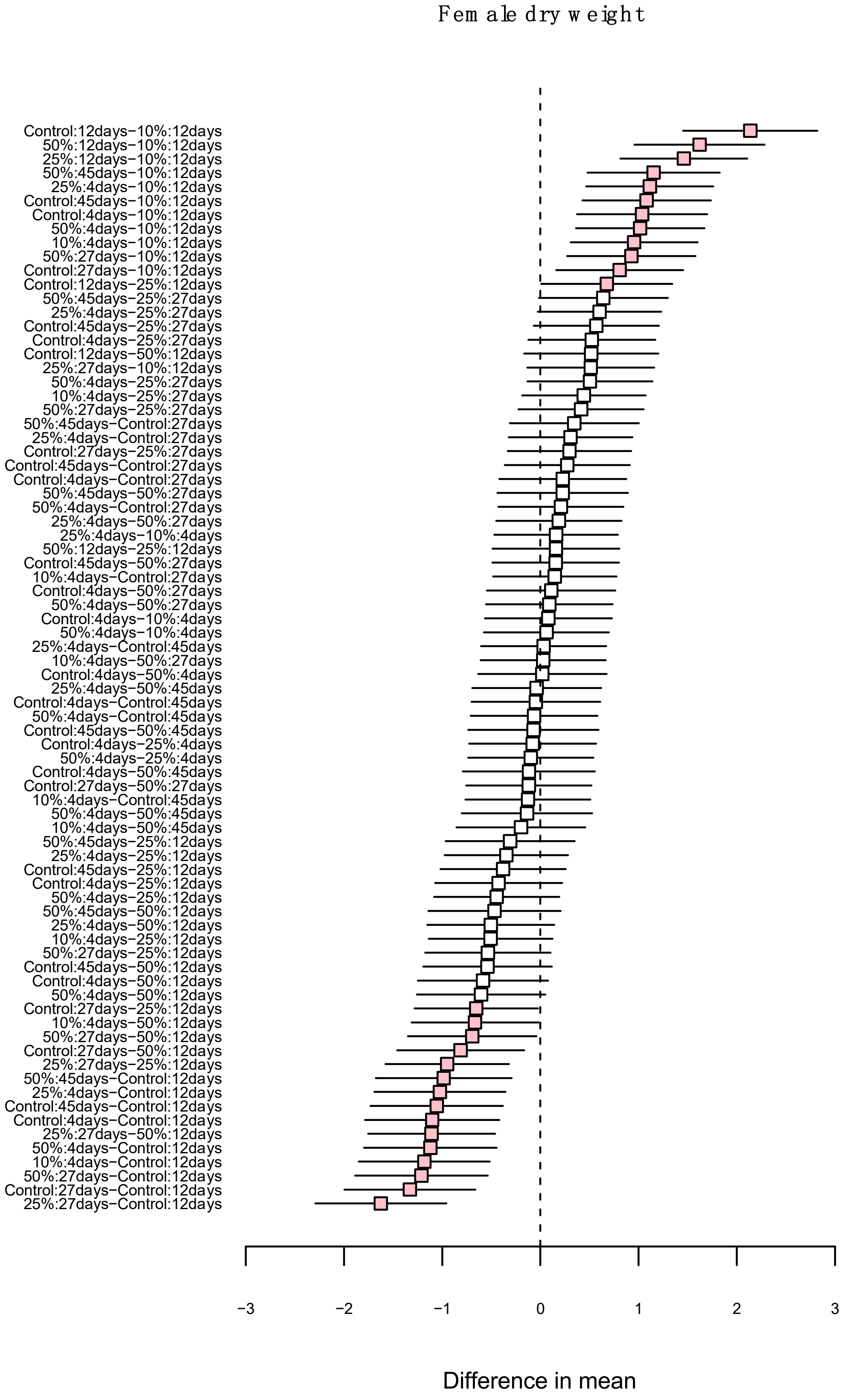

Figure S2. Forrest-plot of Tukey-HSD testing the effects of diet and age on female fly dry weight. All factors were treated as categorical variables. Red indicates statistically significant difference in the pairwise comparison between diets and ages.

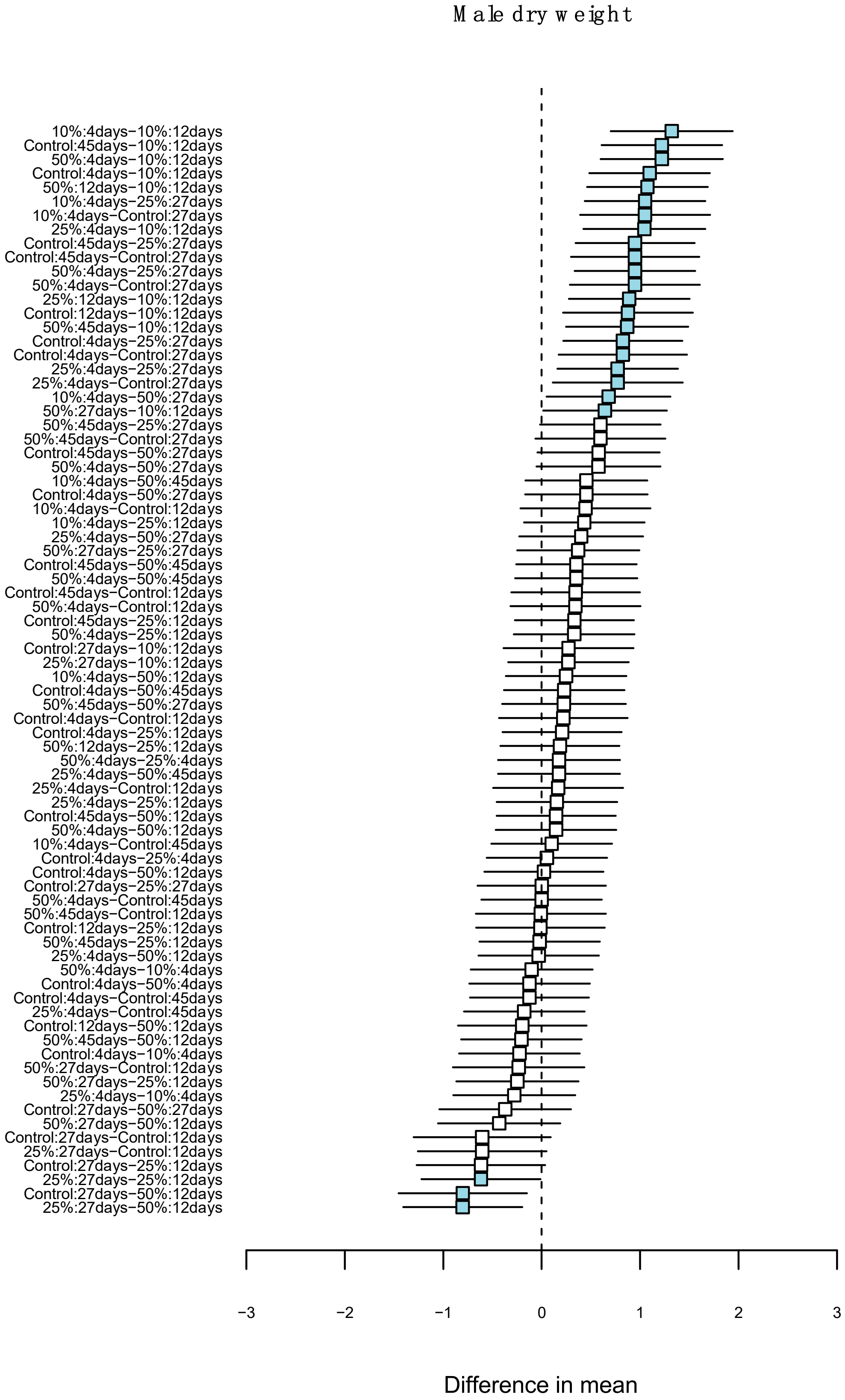

Figure S3. Forrest-plot of Tukey-HSD testing the effects of diet and age on male fly dry weight. All factors were treated as categorical variables. Blue indicates statistically significant difference in the pairwise comparison between diets and ages.

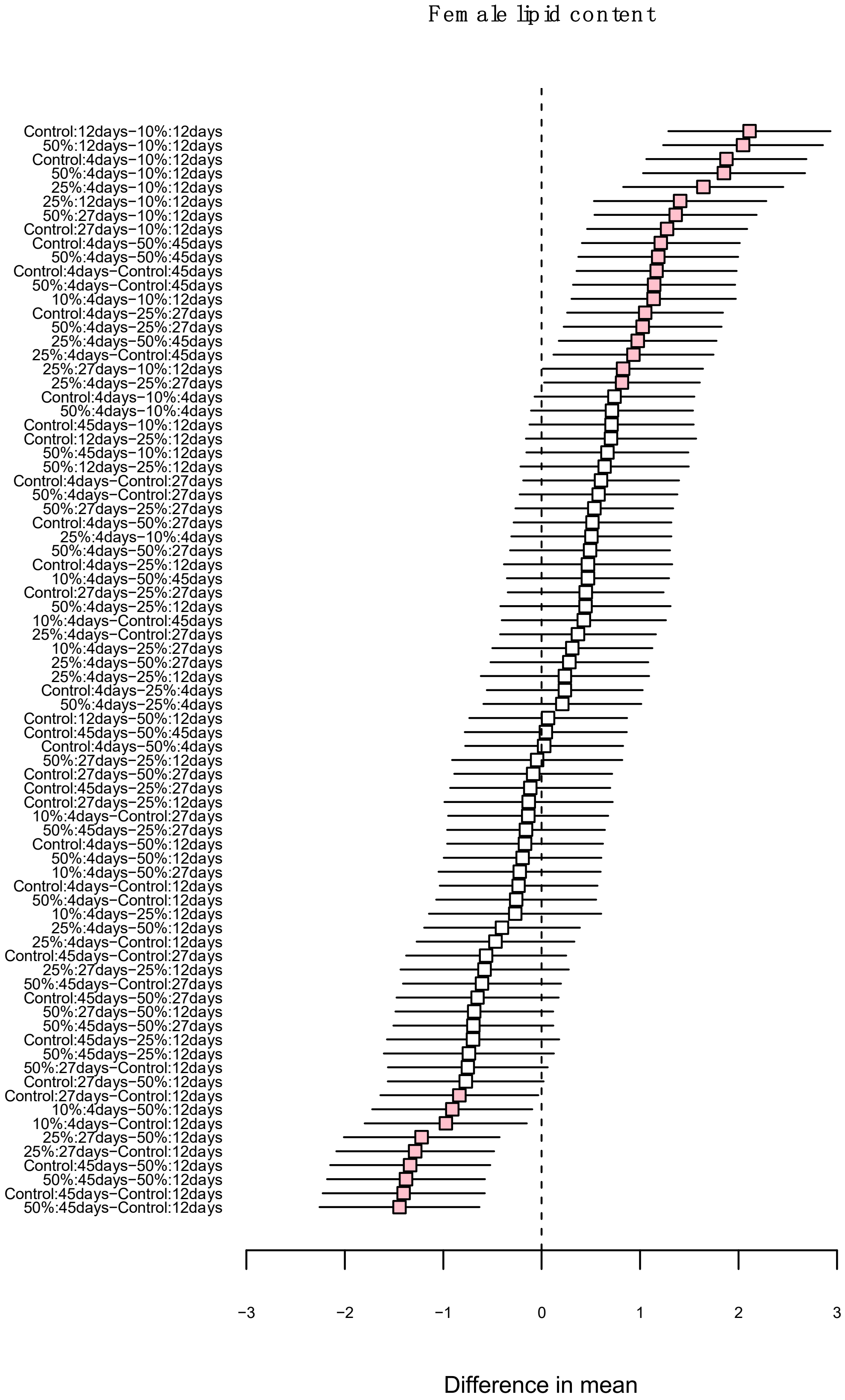

Figure S4. Forrest-plot of Tukey-HSD testing the effects of diet and age on female fly lipid content. All factors were treated as categorical variables. Red indicates statistically significant difference in the pairwise comparison between diets and ages.

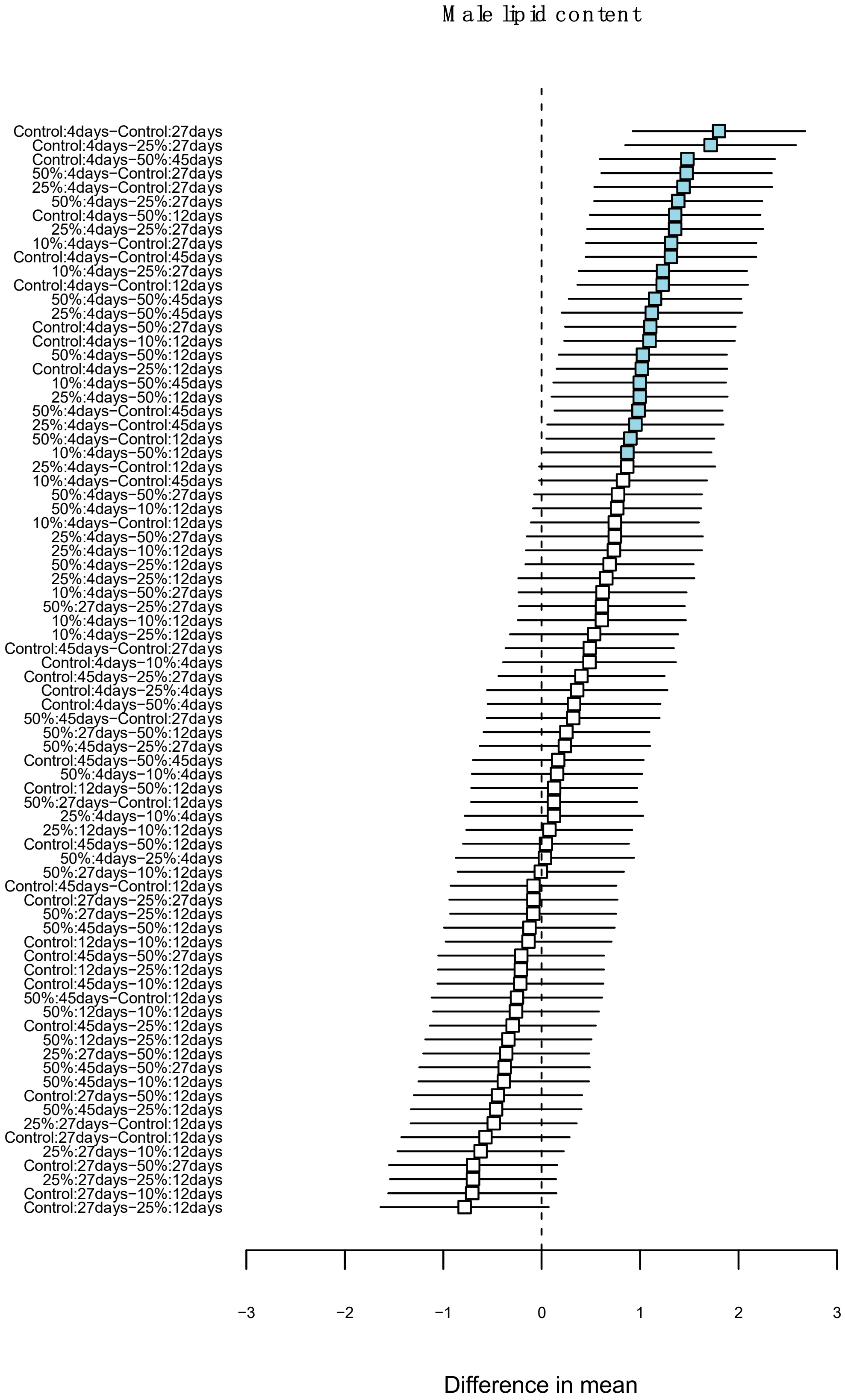

Figure S5. Forrest-plot of Tukey-HSD testing the effects of diet and age on male fly lipid content. All factors were treated as categorical variables. Blue indicates statistically significant difference in the pairwise comparison between diets and ages.

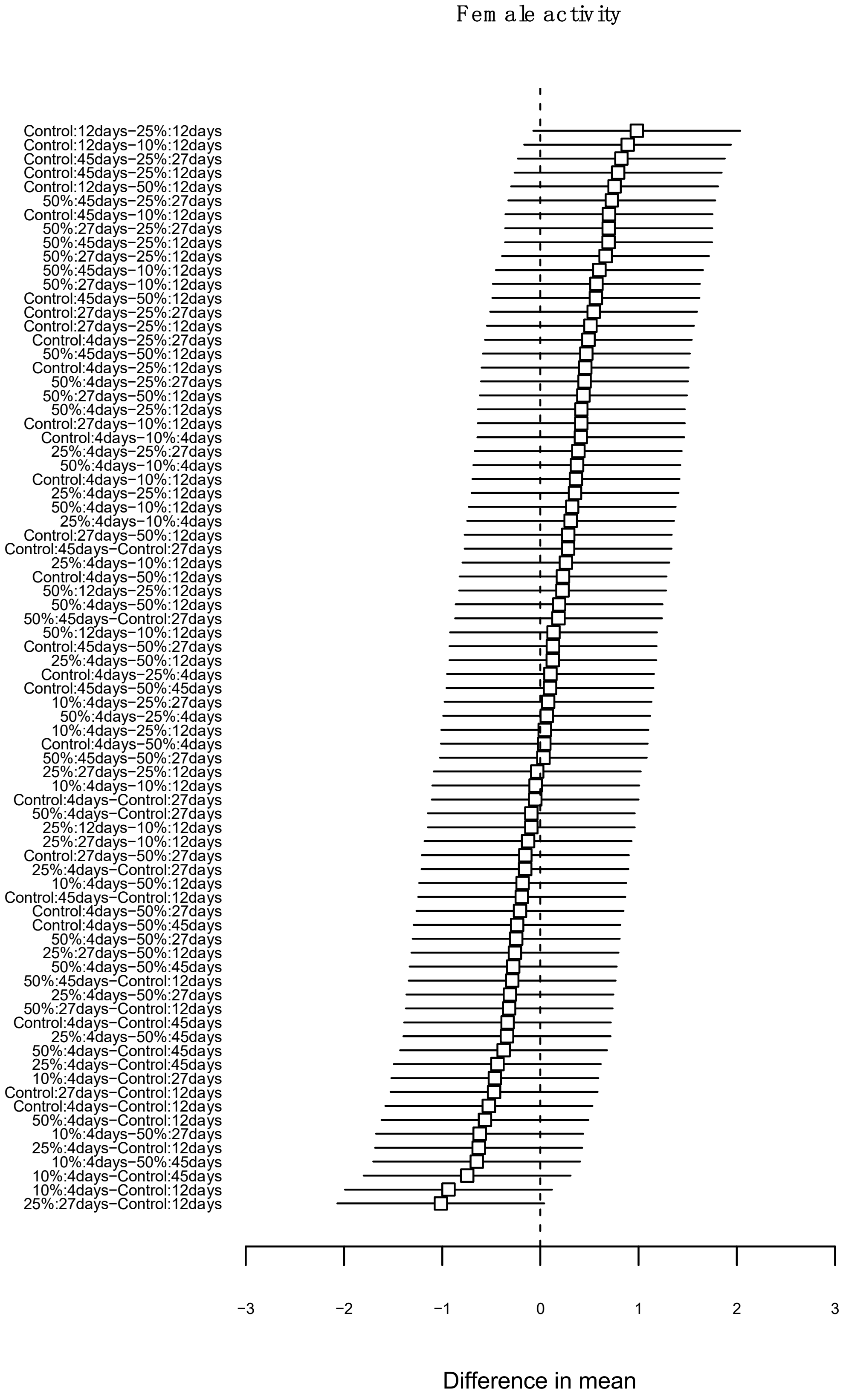

Figure S6. Forrest-plot of Tukey-HSD testing the effects of diet and age on female fly activity. All factors were treated as categorical variables. Red indicates statistically significant difference in the pairwise comparison between diets and ages.

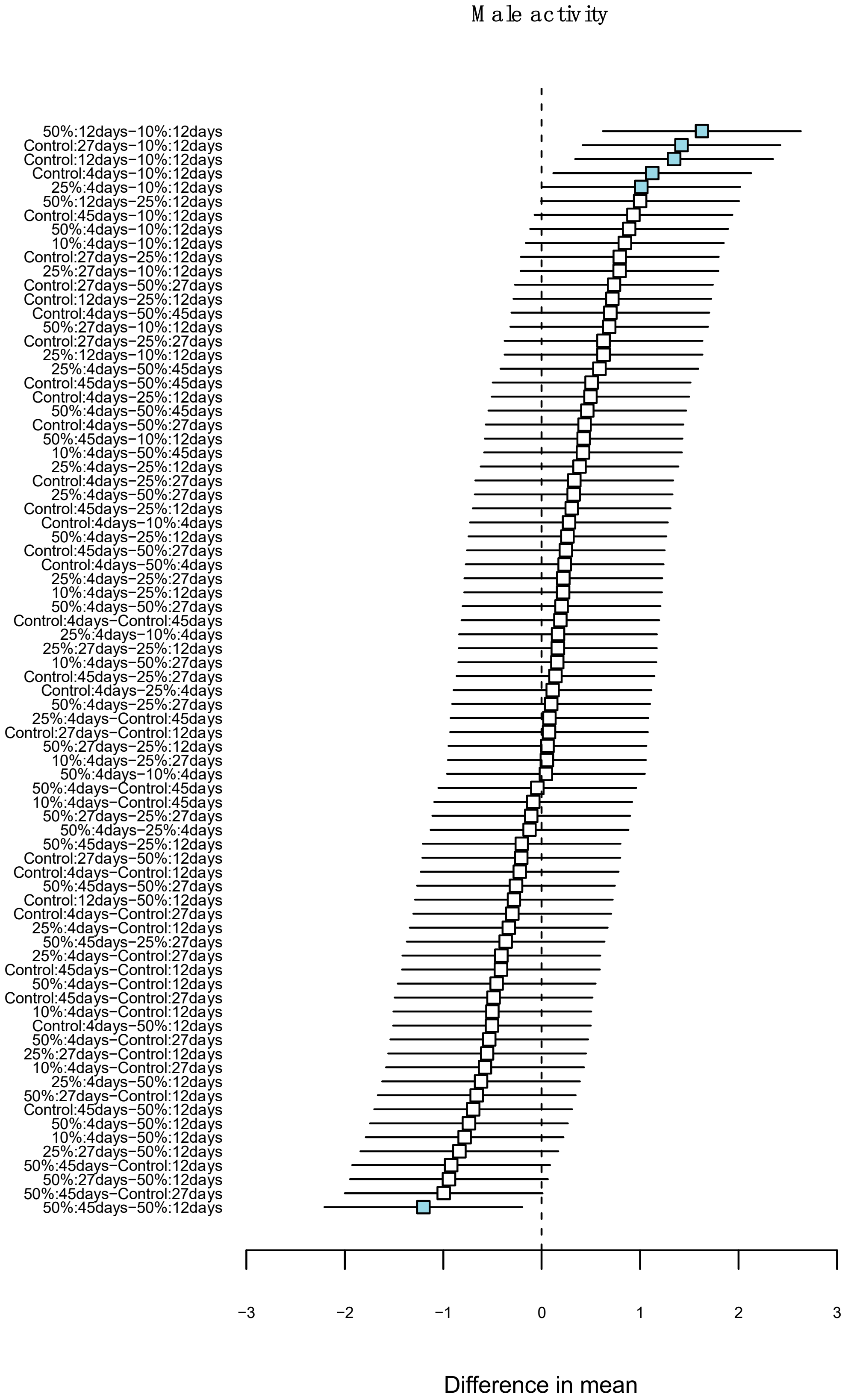

Figure S7. Forrest-plot of Tukey-HSD testing the effects of diet and age on male fly activity. All factors were treated as categorical variables. Blue indicates statistically significant difference in the pairwise comparison between diets and ages.

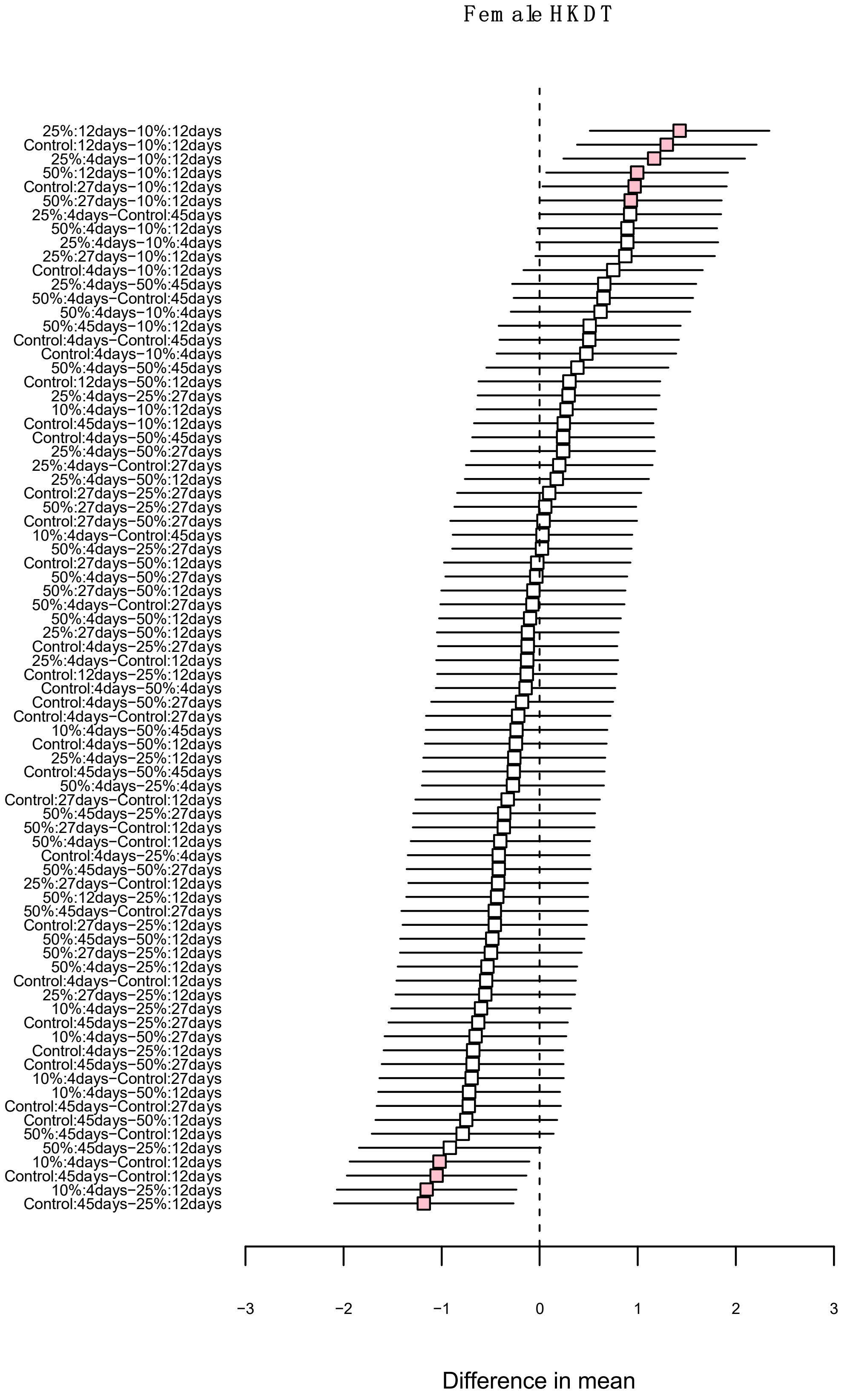

Figure S8. Forrest-plot of Tukey-HSD testing the effects of diet and age on female fly HKDT. All factors were treated as categorical variables. Red indicates statistically significant difference in the pairwise comparison between diets and ages.

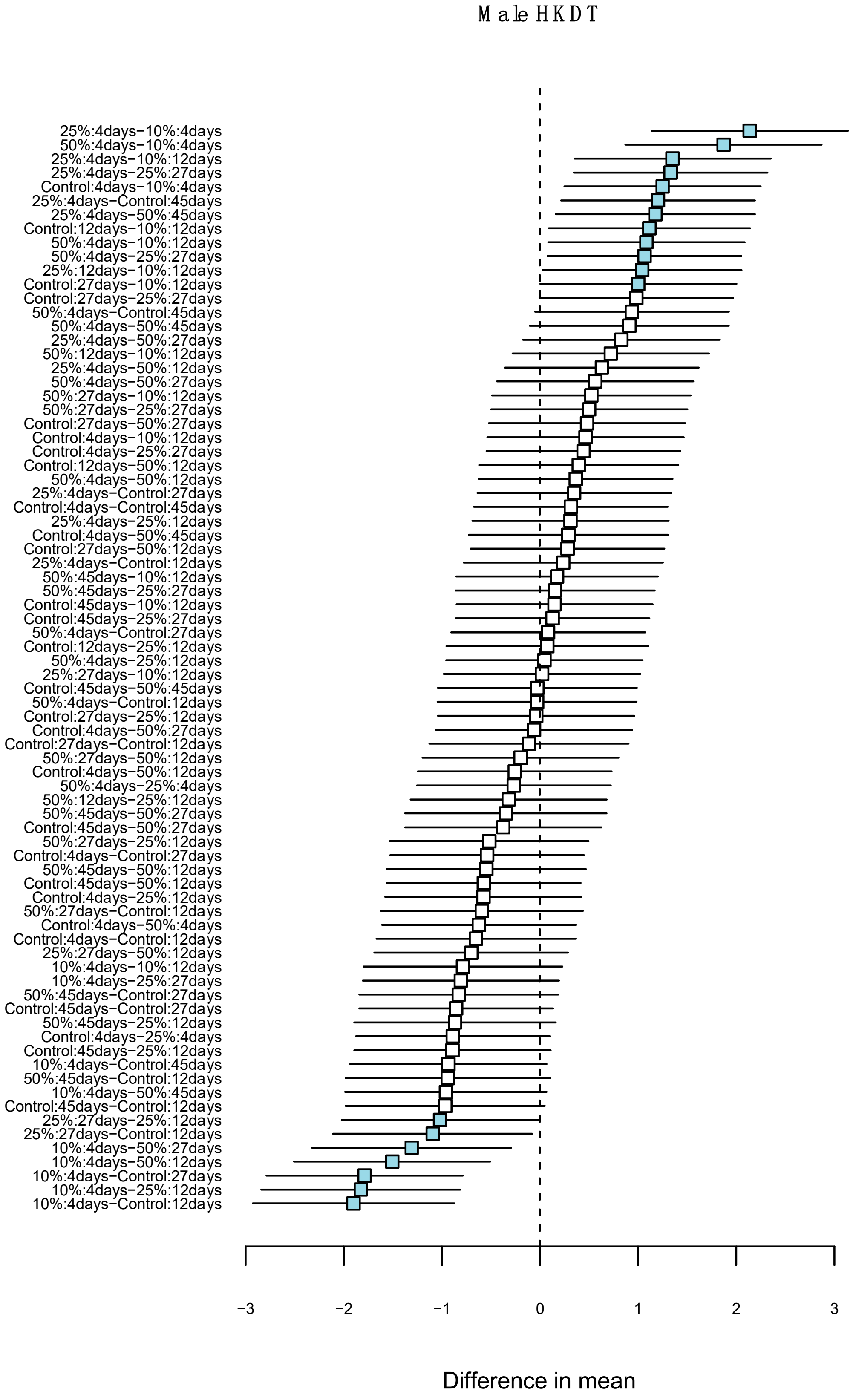

Figure S9. Forrest-plot of Tukey-HSD testing the effects of diet and age on male fly HKDT. All factors were treated as categorical variables. Blue indicates statistically significant difference in the pairwise comparison between diets and ages.

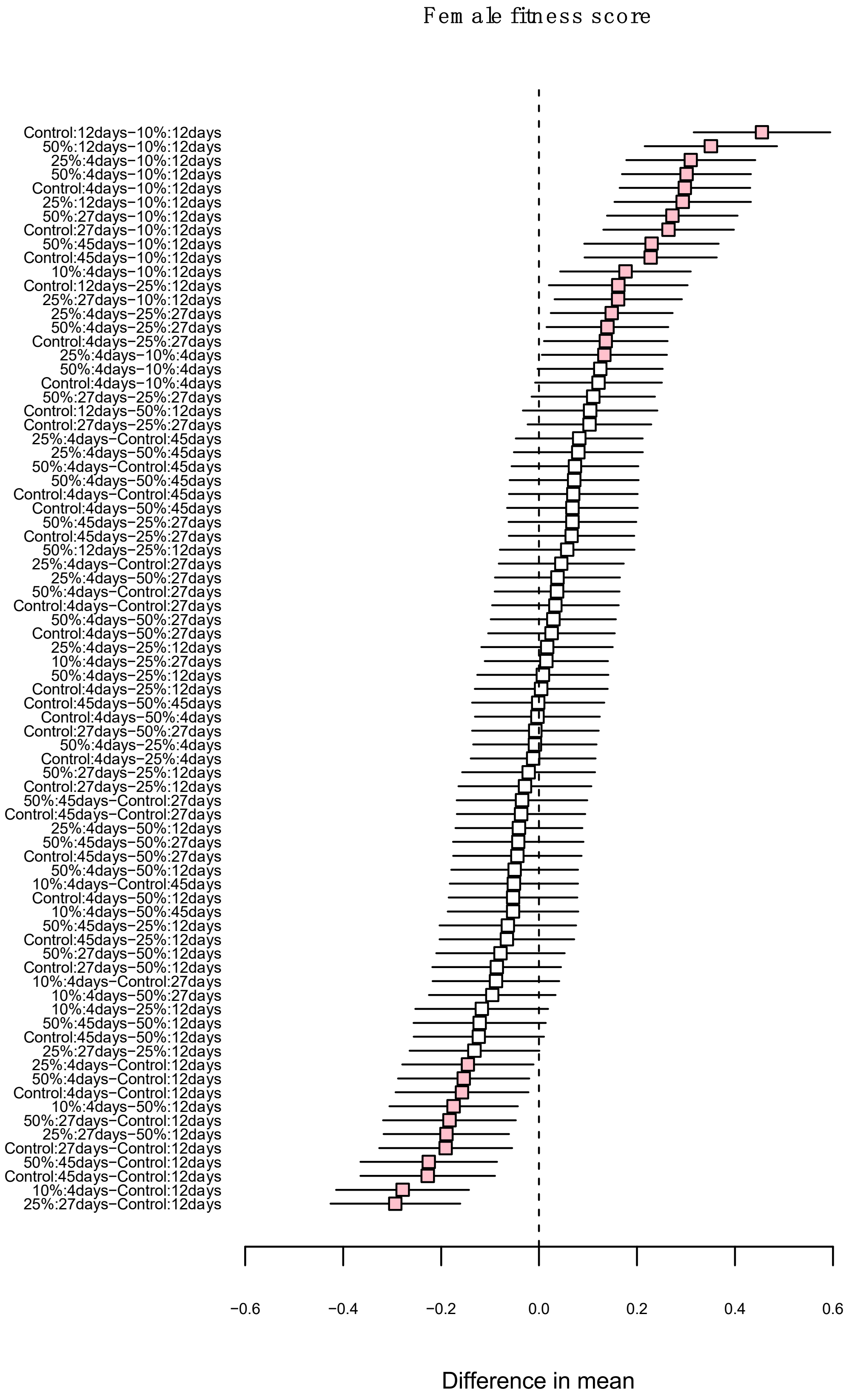

Figure S10. Forrest-plot of Tukey-HSD testing the effects of diet and age on female fly fitness score. All factors were treated as categorical variables. Red indicates statistically significant difference in the pairwise comparison between diets and ages.

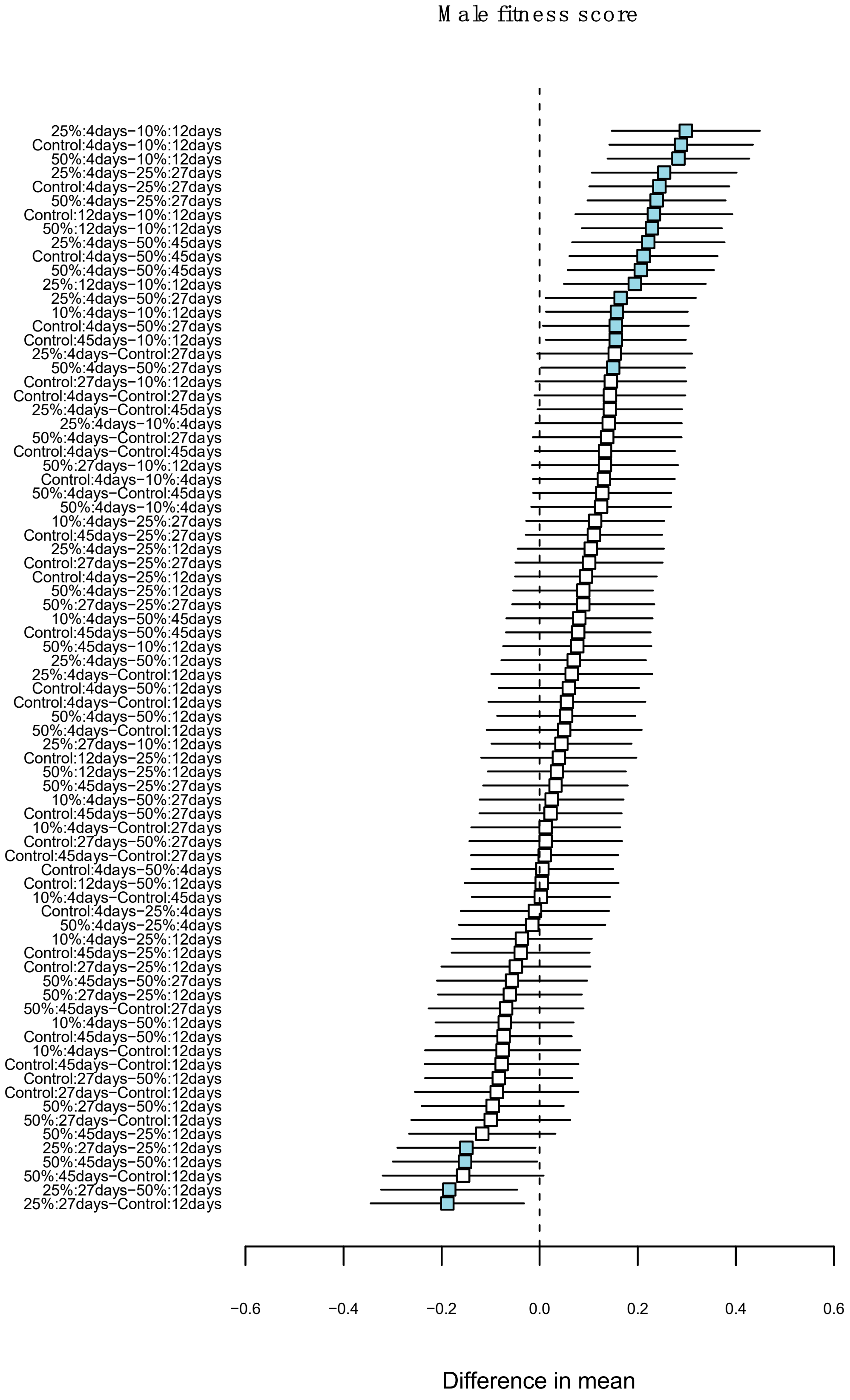

Figure S11. Forrest-plot of Tukey-HSD testing the effects of diet and age on male fly fitness score. All factors were treated as categorical variables. Blue indicates statistically significant difference in the pairwise comparison between diets and ages.

Table S9. Summary of Multivariate cox analysis on lifespan of sex and diet.

	Lifespan					
	**Coef**	**Se(Coef)**	**Lower.95**	**Upper.95**	**z-value**	***P* value**
Male:Female	0.17	0.07	1.03	1.36	2.40	0.016
Male-Control:Male-50%	0.54	0.21	1.15	2.58	2.63	0.009
Male-Control:Male-25%	1.83	0.27	3.70	10.57	6.78	$1.23 \cdot 10^{-11}$
Male-Control:Male-10%	-4.69	0.76	0.002	0.04	-6.21	$5.44 \cdot 10^{-10}$
Female-Control:Female-50%	-0.36	0.21	0.46	1.05	-1.73	0.084
Female-Control:Female-25%	0.67	0.21	1.30	2.94	3.22	0.001
Female-Control:Female-10%	-4.64	0.75	0.002	0.04	-6.16	$7.48 \cdot 10^{-10}$

Table S10. Summary of ANOVA on dry weight testing the effects of sex, diet, age, and all possible interaction effects between these. All factors were treated as categorical variables.

	Dry weight			
	**DF**	**Sum Sq**	**F-value**	***P*-value**
Sex	1	245.7	775.5	$2 \cdot 10^{-16}$
Diet	3	14.8	15.6	$1.08 \cdot 10^{-9}$
Age	3	26.6	28.0	$< 2 \cdot 10^{-16}$
Diet:Sex	3	3.9	4.1	0.006
Diet:Age	6	29.8	15.7	$< 2 \cdot 10^{-16}$
Age:Sex	3	10.8	11.3	$3.44 \cdot 10^{-7}$
Diet:Age:Sex	6	4.6	2.4	0.027
Residuals	461	146.1		

Table S11. Summary of ANOVA on dry weight of female and male flies separately. The ANOVA included diet, age, and all possible interaction effects between these. All factors were treated as categorical variables.

	Dry weight							
	Female				*Male*			
	**DF**	**Sum Sq**	**F-value**	***P*-value**	**DF**	**Sum Sq**	**F-value**	***P*-value**
Diet	3	3.3	15.0	$6.02 \cdot 10^{-9}$	3	14.8	3.6	0.014
Age	3	23.2	18.5	$8.23 \cdot 10^{-11}$	3	18.3	26.4	$3.01 \cdot 10^{-14}$
Diet:Age	6	12.2	11.4	$11.43 \cdot 10^{-11}$	6	22.6	6.7	$1.50 \cdot 10^{-6}$
Residuals	230	70.0			231	76.1		

Table S12. Summary of ANOVA on lipid content testing the effects of sex, diet, age, and all possible interaction effects between these. All factors were treated as categorical variables.

	Lipid content			
	**DF**	**Sum Sq**	**F-value**	***P*-value**
Sex	1	68.3	122.4	$< 2.00 \cdot 10^{-16}$
Diet	3	6.2	3.7	0.011
Age	3	98.4	58.9	$< 2.00 \cdot 10^{-16}$
Diet:Sex	3	22.4	13.4	$2.16 \cdot 10^{-8}$
Diet:Age	6	6.5	1.9	0.073
Age:Sex	3	14.6	8.7	$1.24 \cdot 10^{-5}$
Diet:Age:Sex	6	14.0	4.2	$0.42 \cdot 10^{-3}$
Residuals	468	260.9		

Table S13. Summary of ANOVA on lipid content of female and male flies separately. The ANOVA included diet, age, and all possible interaction effects between these. All factors were treated as categorical variables.

	Lipid content							
	Female				*Male*			
	**DF**	**Sum Sq**	**F-value**	***P*-value**	**DF**	**Sum Sq**	**F-value**	***P*-value**
Diet	3	25.2	16.1	$1.50 \cdot 10^{-9}$	3	2.7	1.5	0.204
Age	3	51.3	32.8	$< 2.00 \cdot 10^{-16}$	3	58.2	32.7	$< 2.00 \cdot 10^{-16}$
Diet:Age	6	11.4	3.6	$1.81 \cdot 10^{-3}$	6	9.4	2.6	0.017
Residuals	232	121.0			236	139.9		

Table S14. Summary of ANOVA on locomotor activity testing the effects of sex, diet, age, and all possible interaction effects between these. All factors were treated as categorical variables.

	Locomotor activity			
	**DF**	**Sum Sq**	**F-value**	***P*-value**
Sex	1	9.8	11.0	$0.96 \cdot 10^{-3}$
Diet	3	31.8	12.0	$1.35 \cdot 10^{-7}$
Age	3	2.0	0.76	0.527
Diet:Sex	3	2.0	0.77	0.512
Diet:Age	6	10.4	2.0	0.069
Age:Sex	3	7.8	2.9	0.033
Diet:Age:Sex	6	15.8	3.0	$7.14 \cdot 10^{-3}$
Residuals	494	436.8		

Table S15. Summary of ANOVA on locomotor activity of female and male flies separately. The ANOVA included diet, age, and all possible interaction effects between these. All factors were treated as categorical variables.

	Locomotor activity							
	Female				*Male*			
	**DF**	**Sum Sq**	**F-value**	***P*-value**	**DF**	**Sum Sq**	**F-value**	***P*-value**
Diet	3	16.8	6.1	$0.54 \cdot 10^{-3}$	3	17.1	6.8	$0.21 \cdot 10^{-3}$
Age	3	1.2	0.44	0.722	3	8.5	3.4	0.019
Diet:Age	6	7.2	1.3	0.261	6	19.1	3.8	$1.30 \cdot 10^{-3}$
Residuals	247	228.8			247	208.0		

Table S16. Summary of ANOVA on HKDT testing the effects of sex, diet, age, and all possible interaction effects between these. All factors were treated as categorical variables.

	HKDT			
	**DF**	**Sum Sq**	**F-value**	***P*-value**
Sex	1	13.0	17.2	$3.96 \cdot 10^{-5}$
Diet	3	62.3	27.5	$< 2 \cdot 10^{-16}$
Age	3	32.3	14.3	$6.40 \cdot 10^{-9}$
Diet:Sex	3	2.4	0.75	0.523
Diet:Age	6	17.8	1.1	0.358
Age:Sex	3	1.7	3.9	$0.74 \cdot 10^{-3}$
Diet:Age:Sex	6	11.8	2.6	0.017
Residuals	480	362.4		

Table S17. Summary of ANOVA on HKDT of female and male flies separately. The ANOVA included diet, age, and all possible interaction effects between these. All factors were treated as categorical variables.

	HKDT							
	Female				*Male*			
	**DF**	**Sum Sq**	**F-value**	***P*-value**	**DF**	**Sum Sq**	**F-value**	***P*-value**
Diet	3	25.1	12.0	$2.43 \cdot 10^{-7}$	3	39.1	16.1	$1.53 \cdot 10^{-9}$
Age	3	12.5	6.0	$5.92 \cdot 10^{-4}$	3	21.4	8.8	$1.52 \cdot 10^{-5}$
Diet:Age	6	5.6	1.3	0.238	6	24.1	4.9	$8.64 \cdot 10^{-5}$
Residuals	241	168.2			239	194.2		

Table S18. Summary of ANOVA on the fitness score testing the effects of sex, diet, age, and all possible interaction effects between these. All factors were treated as categorical variables.

	Fitness score			
	**DF**	**Sum Sq**	**F-value**	***P*-value**
Sex	1	2.37	165.4	$< 2 \cdot 10^{-16}$
Diet	3	1.49	34.7	$< 2 \cdot 10^{-16}$
Age	3	1.74	40.5	$< 2 \cdot 10^{-16}$
Diet:Sex	3	0.12	2.8	0.039
Diet:Age	6	0.55	6.4	$1.88 \cdot 10^{-6}$
Age:Sex	3	0.19	4.5	0.004
Diet:Age:Sex	6	0.16	1.8	0.096
Residuals	430	6.15		

Table S19. Summary of ANOVA on the fitness score of female and male flies separately. The ANOVA included diet, age, and all possible interaction effects between these. All factors were treated as categorical variables.

	Fitness score							
	Female				*Male*			
	**DF**	**Sum Sq**	**F-value**	***P*-value**	**DF**	**Sum Sq**	**F-value**	***P*-value**
Diet	3	1.15	30.6	$< 2 \cdot 10^{-16}$	3	0.39	8.1	$3.67 \cdot 10^{-5}$
Age	3	0.57	15.2	$5.20 \cdot 10^{-9}$	3	1.35	28.1	$2.16 \cdot 10^{-15}$
Diet:Age	6	0.54	7.3	$4.65 \cdot 10^{-7}$	6	0.16	1.6	0.143
Residuals	213	2.66			217	3.49		
